# CarieCheck: An mHealth App for Caries-Risk Self-Assessment—User-Perceived Usability and Quality in a Pilot Study

**DOI:** 10.3390/dj14010031

**Published:** 2026-01-05

**Authors:** Eduardo Guerreiro, Guilherme Souza, José João Mendes, Ana Cristina Manso, João Botelho

**Affiliations:** Egas Moniz Center for Interdisciplinary Research (CiiEM), Egas Moniz School of Health & Science, 2829-511 Almada, Portugaljmendes@egasmoniz.edu.pt (J.J.M.); cmanso@egasmoniz.edu.pt (A.C.M.); jbotelho@egasmoniz.edu.pt (J.B.)

**Keywords:** mHealth, mobile applications, caries risk assessment, preventive dentistry, artificial intelligence, digital health, MARS, oral health

## Abstract

**Background/Objectives:** Mobile health (mHealth) technologies are increasingly used to support preventive oral care and patient self-management. CarieCheck is a Portuguese app intended to improve oral health literacy and support caries-risk self-assessment. This prospective pilot study focused on users’ perceived app quality and usability, assessed with uMARS-PT. **Methods**: Thirty participants from the academic community of Egas Moniz School of Health and Science used the app for 30 days and completed the uMARS-PT questionnaire. Descriptive statistics were used to calculate mean scores for Engagement, Functionality, Aesthetics, Information Quality, Subjective Quality, and Perceived Impact. **Results:** The overall mean uMARS-PT score was 4.22, indicating excellent perceived quality. The highest domain scores were Functionality (4.51), Aesthetics (4.45), and Information Quality (4.22). Engagement (3.71) and Subjective Quality (3.05) were moderate. Perceived Impact (3.85) reflected self-reported perception of increased awareness and motivation regarding oral health behaviors. **Conclusions:** CarieCheck was rated highly in usability, aesthetics, and information quality. These findings suggest that CarieCheck may be considered as a digital tool for preventive education and user-supported caries-risk self-assessment. Larger, longer-term studies in diverse populations using objective behavioral and clinical outcomes are warranted.

## 1. Introduction

Dental caries remains one of the most prevalent diseases worldwide, affecting approximately 2.3 billion people with permanent teeth [1]. This condition arises from an imbalance in the oral biofilm ecosystem, largely triggered by the frequent consumption of fermentable carbohydrates [2,3]. Fluctuations in oral pH drive alternating cycles of demineralization and remineralization; when demineralization predominates, irreversible loss of tooth structure can occur. Without timely management, lesions may progress towards the dentine–pulp interface, causing pain and discomfort. Dental caries is therefore associated with reduced quality of life and a substantial economic burden for individuals and healthcare systems [4,5]. As caries advances, individuals may experience chewing difficulties, pain, and tooth loss; in children, it can contribute to delayed speech development and increased school absenteeism, while in adults it is associated with work absenteeism [6,7].

Because plaque bacteria drive caries progression, daily plaque control—via toothbrushing and interdental cleaning—is essential. Plaque buildup is associated with higher risk of caries and periodontal disease and may contribute to oral inflammation and accelerated alveolar bone loss, potentially leading to premature tooth loss [8]. Preventive success also depends on patients’ engagement, since adherence is influenced by motivation, self-efficacy, and ongoing education [9]. Digital health technologies can provide structured support for these behaviors and help sustain preventive routines over time [10,11].

According to the World Health Organization (WHO), eHealth refers to the use of information and communication technologies in health, including electronic health records, telemedicine, and digital education platforms. Within eHealth, mobile health (mHealth) refers to the use of mobile devices, such as smartphones, tablets, and wearable sensors, to deliver health services and information in real time. These tools can help users track risk factors, receive personalized feedback, and support engagement in preventive care [12,13].

Recent evidence suggests that, when properly designed and validated, mobile applications can be valuable tools for reducing oral health disparities, improving access to information, and fostering a user-centered, participatory approach to healthcare [14,15,16].

Building on this digital transition, mobile applications have increasingly been explored as tools to support self-management and preventive oral care. One example is CarieCheck, a mobile application developed within the academic environment of the Egas Moniz School of Health and Science aimed at promoting oral health awareness and supporting self-assessment and self-management regarding caries prevention. The app provides users with structured questionnaires and educational feedback based on individual risk profiles, seeking to empower them to make informed decisions about their daily oral health routines.

Given the increasing integration of mobile health technologies into preventive oral care, it is essential to assess the scientific quality, usability, and effectiveness of such ap-plications before recommending them to the general population. Despite the growing number of oral health–related apps available in app stores, only a small proportion have been evaluated using standardized, validated instruments such as the Mobile App Rating Scale (MARS) or uMARS-PT [17,18].

Moreover, there is a lack of evidence exploring the perception, engagement, and satisfaction of users regarding mobile oral health applications within the Portuguese context.

Accordingly, this pilot study aimed to evaluate the CarieCheck mobile application using uMARS-PT (Portuguese user Mobile App Rating Scale; an end-user adaptation of MARS) in a sample from the academic community of Egas Moniz School of Health and Science. The results provide preliminary, user-centered evidence on perceived app quality across engagement, functionality, aesthetics, and information quality, relevant to preventive oral health education and self-management. This study addressed the following research question: Is CarieCheck perceived by users as engaging, functional, and informative? Based on MARS guidance, we considered mean domain scores ≥3.0 to indicate acceptable quality.

## 2. Materials and Methods

This was a feasibility and usability pilot study, not designed to establish causal relationships. It was adopted a prospective, observational, descriptive and cross-sectional design with a pilot character, aiming to evaluate the mobile application CarieCheck using the uMARS-PT, validated for Portuguese [19]. The study was conducted in accordance with the ethical principles of the Declaration of Helsinki (1975, revised in 2013) and was approved by the Ethics Committee of Egas Moniz School of Health and Science (ID no. 1462 PT 257/24; Approval Date: 27 November 2024).

Participation was entirely voluntary, and all participants provided written informed consent prior to inclusion. Anonymity and data confidentiality were ensured throughout the study, in compliance with the General Data Protection Regulation (GDPR).

### 2.1. Study Setting and Participants

The sample was selected by convenience from the academic community of Egas Moniz School of Health and Science, which includes students, faculty members, administrative staff, and oral health professionals working at the Egas Moniz University Clinic—Caparica. This community was chosen because it allowed the inclusion of diverse population groups within a controlled and familiar environment, and because, at the time of the study, the application was a test version awaiting publication of its patent registration. A total of 30 participants were included in this pilot study, a sample size considered appropriate for feasibility-focused designs. According to current methodological guidelines, pilot studies aiming to test procedures or acceptability may adequately operate with samples of around 30 participants, as they are not intended to estimate effect sizes or perform inferential analyses [20,21].

Participants were invited to install and use the test version of the CarieCheck app for a 30-day period. During the study period, the CarieCheck app was tested using a pre-release version available for both iOS and Android platforms. The decision to adopt a 30-day trial window is supported by previous mHealth feasibility research, where similar 30-day intervals have been used to assess self-perceived acceptability, usability, adherence patterns, and behavioral engagement with digital health interventions. This duration provides enough time for repeated interaction and real-world usage, while avoiding excessive participant burden [22]. After the trial period, participants were asked to complete an online questionnaire created using the Qualtrics XM platform, which included uMARS-PT for app quality assessment.

App usage was monitored through the administrator dashboard of the CarieCheck test version. This internal panel allowed the research team to verify, in real time, whether participants were completing the in-app questionnaires and interacting with the core features. Although individual usage metrics were not quantitatively analyzed, this monitoring confirmed active engagement throughout the 30-day trial period.

Inclusion criteria were age ≥ 18 years, affiliation with the Egas Moniz academic community, ownership of a compatible smartphone, and provision of written informed consent.

Exclusion criteria included an age under 18, non-affiliation with the Egas Moniz academic community, the lack of a compatible smartphone, or failure to sign the informed con-sent form [20,21].

### 2.2. CarieCheck App

The CarieCheck mobile application is a digital tool developed to facilitate dental caries risk assessment and to support monitoring and management over time. Developed by researchers at Egas Moniz School of Health and Science, the app integrates scientific evidence with an interactive and educational approach aimed at empowering users to identify and understand their individual caries risk through a user-friendly interface that combines behavioral, clinical and lifestyle factors. Its primary goal is to enhance user’s oral health literacy and encourage sustained engagement in preventive dental care.

The system is structured around four core components: (1) a personalized caries risk assessment tool based on scientifically validated parameters; (2) a daily monitoring interface supported by gamification principles to motivate users to adopt and maintain positive oral hygiene habits; (3) a secure communication module that enables remote interaction between patients and oral health professionals; and (4) automatic generation of individualized digital reports summarizing risk levels and tailored recommendations using artificial intelligence. This multidimensional design is intended to support preventive education for the public and to complement clinical workflows, aligning digital health technology with patient-centered care. At the time of this evaluation, the pre-release version tested included the core functionalities for caries-risk self-assessment and educational feedback. Artificial-intelligence-based features were under development and were not evaluated in the present pilot study.

**Figure 1:**
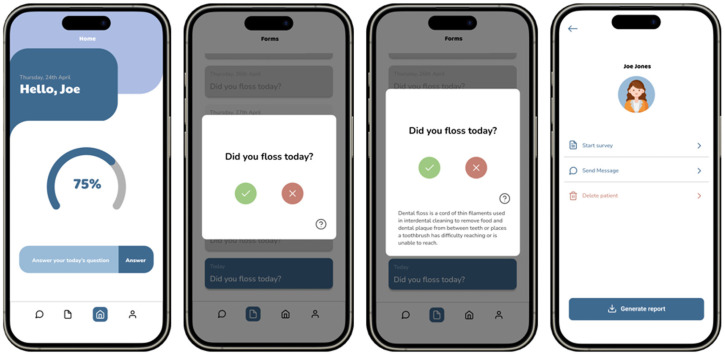
Example of the CarieCheck mobile application interface. The app provides users with daily interactive questions related to oral health habits, such as flossing, and delivers immediate feedback, progress tracking, and personalized communication between patients and dental professionals.

CarieCheck App (Figure 1) was designed to address some practical limitations of traditional caries-risk models (e.g., CAMBRA, Cariogram^®^), particularly regarding user engagement, accessibility, and continuous self-monitoring, rather than to replace or outperform these models from a clinical predictive standpoint.

Its framework aligns with the eHealth and mHealth frameworks recommended by the World Health Organization, offering a solution that can be integrated into public health strategies and routine clinical practice. The application is registered in the European Patent Bulletin no. 2025/36 under publication number 4 610 990, underscoring its originality and the potential for technological transfer. Developed in Portugal, CarieCheck may represent a scalable digital approach to support preventive education and user engagement, integrating behavioral principles and digital technology within an accessible platform.

### 2.3. uMARS-PT Questionnaire

The evaluation of the CarieCheck mobile application was conducted using the uMARS-PT, a validated and widely used multidimensional instrument designed to assess the quality of health-related mobile applications. Developed by Stoyanov and colleagues, the original MARS comprises 23 items organized into five domains: Engagement, Functionality, Aesthetics, Information Quality, and Subjective Quality. Each item is rated on a 5-point Likert scale (1 = Inadequate; 2 = Poor; 3 = Acceptable; 4 = Good; 5 = Excellent), with non-applicable (N/A) items excluded from domain analysis [23,24].

For this study, the Portuguese-validated version of the MARS (uMARS-PT) was used [19]. This version includes three additional questions designed to capture user’s perceived impact and behavioral intention related to health-promoting practices. Participants completed the online questionnaire via Qualtrics XM after a 30-day trial period using the CarieCheck app. For each domain, mean scores were calculated by averaging the non-missing item values. The overall uMARS-PT score was computed as the arithmetic mean of the four objective domains (Engagement, Functionality, Aesthetics, and Information Quality), following the procedure established in the validation protocol. The Subjective Quality and additional Perceived Impact domains were analyzed separately to provide further insight into user satisfaction and behavioral influence.

In this context, the term “behavioral impact” does not refer to objectively measured behavior change, but rather to participants’ self-reported perceived impact. Specifically, the uMARS-PT items capture whether users felt more aware, motivated, or inclined to adopt healthier oral health behaviors because of interacting with the app. These items therefore assess perceived influence rather than actual behavioral outcomes. Interpretation followed the standard uMARS-PT classification: mean scores ≥ 4.0 indicated excellent perceived quality; 3.0–3.9, acceptable/moderate quality; and <3.0, poor or unsatisfactory quality.

### 2.4. Statistical Analysis

Data analysis was performed using IBM SPSS Statistics version 28.0 (IBM Corp., Armonk, NY, USA). A descriptive statistical analysis was conducted to summarize participants responses to the uMARS-PT. For each of the four objective uMARS-PT domains (Engagement, Functionality, Aesthetics, and Information Quality), mean scores and standard deviations were calculated. The overall uMARS-PT score was determined as the arithmetic mean of these four domains, in accordance with the original validation protocol. The Subjective Quality domain was analyzed separately to provide additional insight into user satisfaction and perceived app usability.

No inferential tests were performed, as the study aimed to provide an exploratory, descriptive evaluation of user perceptions in a pilot context.

## 3. Results

### 3.1. Participants Inclusion and Characteristics

A total of 30 participants were included in this pilot study, all recruited by convenience from the academic community of Egas Moniz School of Health and Science, which comprises students, faculty members, administrative staff, and oral health professionals. The sample was intentionally diverse to include individuals of different professional roles and genders within a familiar and controlled environment. No participants were excluded from the analysis, as all those enrolled completed the 30-day trial period and submitted valid responses to the uMARS-PT questionnaire.

Among the 30 participants, 23 (76.7%) were students—15 females (65.2%) and 8 males (34.8%). In addition, two (6.7%) were male professors, one (3.3%) was a male dental intern, one (3.3%) was a male Volunteer Dentist in Training, and three (10%) were female administrative staff as shown in Table 1.

### 3.2. Descriptive Analysis of uMARS-PT Domains

Table 2 presents the descriptive analysis of all uMARS-PT items, while domain-level results are summarized to highlight overall patterns in perceived app quality. The mean scores for each item, as well as the minimum, maximum, and standard deviation values, are displayed to illustrate users’ overall evaluation of the CarieCheck mobile application.

Across the 26 items, the mean scores ranged between 2.30 and 4.67 on a five-point Likert scale. The highest individual mean score corresponded to the Ease-of-Use item (Mean = 4.67 ± 0.55), whereas the lowest score was observed for Disposition to Pay (Mean = 2.30 ± 1.06). Items related to Functionality and Aesthetics domains generally achieved higher mean values compared with other domains. Lower mean values were mainly found in the Engagement and Subjective Quality domains. Overall, the obtained results suggest consistent variability across domains, reflecting different aspects of the users experience with the CarieCheck application.

Table 3 summarizes the mean scores obtained for each uMARS-PT domain and the corresponding qualitative classification.

## 4. Discussion

This pilot, observational, descriptive, and cross-sectional study aimed to evaluate the perceived quality of the CarieCheck mobile application using the uMARS-PT. Conducted among 30 participants from the academic community of Egas Moniz School of Health and Science, the study provided a multidimensional assessment of the app after a 30-day trial period. The overall uMARS-PT score was 4.22, indicating excellent perceived quality. The highest domain scores were observed for Functionality (4.51), Aesthetics (4.45), and Information Quality (4.22), suggesting that users recognized the app’s technical efficiency, appealing visual design, and accurate, relevant content. In contrast, Engagement (3.71), Perceived Impact (3.85), and particularly Subjective Quality (3.05) obtained lower scores, reflecting limited personalization, moderate perceived behavioral influence, and reduced willingness to pay for or highly recommend the app.

The uMARS-PT instrument was selected for this evaluation due to its strong evidence of reliability and validity in assessing mHealth applications. The initial validation of the MARS reported excellent internal consistency (Cronbach’s α ≈ 0.90), supporting its reliability as a measure of global app quality [24]. Subsequent validation work confirmed the four-factor structure of the uMARS-PT across 15 international studies involving 1299 health applications, reporting high reliability coefficients (Omega = 0.79–0.93) and strong in-ter-rater agreement (ICC = 0.82). The widespread cross-cultural validation of the scale reinforces its suitability for studies such as the present one, ensuring comparability and methodological rigor [25].

Regarding individual domains, the Engagement domain (Mean = 3.71) indicated that CarieCheck achieved a moderate-to-good capacity to sustain users’ attention and interest. Previous studies consistently report this as one of the lowest-performing uMARS-PT domains across health apps—mean engagement scores of 2.9 to 3.2 are common [26,27,28]. Therefore, the CarieCheck result aligns with the general trend, suggesting that engagement features—such as reminders, gamification, or adaptive feedback—could be further strengthened.

The Functionality domain obtained the highest mean score (4.51), highlighting an intuitive interface, smooth navigation, and the absence of major technical issues. These results are comparable to or higher than those reported in similar evaluations of nursing or maternal health apps (means = 4.5–5.0) [26,28].

Similarly, the Aesthetics score (4.45) confirmed the app’s strong visual appeal, aligning with evidence that visually coherent and appealing interfaces enhance user satisfaction and experience in mobile applications, including mHealth contexts [29,30].

The Information Quality domain (4.22) also showed a strong performance, suggesting that users perceived the content as clear, reliable, and evidence-based. Similar findings have been reported in evaluations of other mHealth applications, particularly in nursing and diabetes management, where well-structured, scientifically supported content was a key determinant of perceived quality [26]. In the context of oral health, this aspect is especially relevant, as previous reviews have highlighted recurring gaps in the accuracy and transparency of app content [16,31].

The Subjective Quality score (3.05) indicated moderate satisfaction, a common outcome in pilot evaluations where users recognize potential but expect further refinement in features and content. Conversely, the Perceived Impact score (3.85) reflected self-reported perceptions of increased awareness and intention to adopt healthier oral-hygiene behaviors. Comparable discrepancies between satisfaction and perceived benefit have been reported in other mHealth assessments, reflecting the learning and optimization stages typical of early-phase digital interventions [27,28].

Despite these encouraging results, this study has limitations. The small sample size (n = 30), typical of pilot designs, limits generalizability to broader populations. In addition, the 30-day trial may have been insufficient to capture longer-term engagement, retention, or sustained perceived impact. Future studies should therefore include larger and more diverse samples, longer follow-up periods, and formal assessments of continued use.

The exclusive recruitment from an academic health-science environment, likely associated with higher oral health literacy and familiarity with preventive concepts, further limits external validity and may have influenced usability and information-quality ratings, introducing a potential risk of selection bias.

Importantly, this work represents an initial, user-centered phase focused on perceived app quality, usability, and design, assessed using uMARS-PT to inform iterative refinement of CarieCheck. Accordingly, the study did not evaluate objective behavioral changes (e.g., oral hygiene practices) or clinical outcomes such as caries incidence or validated risk indices. The uMARS Perceived Impact domain reflects users’ self-reported awareness and motivation rather than measured behavioral or clinical effects; therefore, conclusions regarding clinical effectiveness cannot be drawn from the present data. Following optimization based on these findings, future research should evaluate CarieCheck in clinical and community settings using objective behavioral endpoints and clinically relevant outcomes.

Additionally, the involvement of some authors in the app’s conceptual development, together with the evaluation within the same institution, may have introduced a risk of positive bias, although independent data collection and analysis procedures were adopted.

Based on these findings, future development could prioritize greater personalization, timely feedback, and engagement-supporting features to improve user experience and sustained use. Further validation across educational, clinical, and community contexts—potentially alongside preventive programs or digital health systems—may help clarify its utility as a tool for oral health promotion and patient self-management.

## 5. Conclusions

This pilot study found that the CarieCheck mobile application achieved a high overall perceived quality rating (mean = 4.22) on the uMARS-PT. In line with the study objectives, CarieCheck was perceived by users as functional, aesthetically appealing, and informationally adequate, with all objective domains exceeding the predefined acceptability threshold (≥3.0). The highest scores were observed in Functionality (4.51), Aesthetics (4.45), and Information Quality (4.22), indicating good usability, interface design, and content quality.

Engagement (3.71) and Subjective Quality (3.05) were comparatively lower, a pattern commonly reported in early-stage mHealth tools, where core usability often develops before sustained user engagement. The Perceived Impact score (3.85) suggested self-reported gains in awareness and motivation regarding oral health behaviors, which may support CarieCheck’s role in preventive education.

Overall, these findings suggest that CarieCheck may be a useful digital tool for preventive oral health education and user-supported caries-risk self-management, within the context of a pilot usability evaluation. Larger longitudinal studies including diverse populations and objective outcomes are needed to assess clinical relevance, retention, and long-term impact.

## 6. Patents

This work resulted in a patent application recently published in the European Patent Bulletin under the publication number EP 4 610 990.

## Figures and Tables

**Table 1:** Participants’ demographic and occupational characteristics (n = 30).

Category	n	%	Gender (n/%)	
			Female	Male
Students	23	76.7	15 (65.2)	8 (34.8)
Professors/Collaborators	2	6.7	0	2
Dental interns	1	3.3	0	1
Volunteer Dentist in Training	1	3.3	0	1
Administrative Staff	3	10.0	3	0
Total	30	100	18 (60)	12 (40)

**Table 2:** Descriptive analysis of all domains of the Mobile App Rating Scale (n = 30).

Item	Engagement Domain	n	Min	Max	Mean	SD
1	Interest: Is the app interesting to use? Does it present information in an engaging way compared with similar apps?	30	1	5	3.9	1.094
2	Entertainment: Is the app fun or enjoyable to use? Does it include features that make it more entertaining than similar apps?	30	1	5	3.59	0.983
3	Customization: Does the app allow users to personalize settings and preferences (e.g., sound, content, notifications)?	30	1	5	3.03	1.033
4	Interactivity: Does the app allow user input, provide feedback, or include prompts (reminders, sharing options, notifications, etc.)?	30	1	5	3.60	0.814
5	Target Group: Is the app’s content (visuals, language, design) appropriate for the intended target group?	30	3	5	4.43	0.679
	**Functionality Domain**					
6	Performance: How accurately and quickly do the app’s features (functions) and components (buttons/menus) operate?	30	3	5	4.57	0.626
7	Ease of Use: How easy is it to learn how to use the app? How clear are the menu tabs, icons, and instructions?	30	3	5	4.67	0.547
8	Navigation: Is movement between screens logical and consistent? Does the app include all necessary links between sections?	30	1	5	4.27	0.868
9	Gestural Design: Do touch, tap, pinch, and scroll gestures make sense? Are they consistent across components and screens?	30	3	5	4.53	0.626
	**Aesthetics Domain**					
10	Layout: Are the arrangement and size of buttons, icons, menus, and on-screen content appropriate?	30	3	5	4.57	0.626
11	Graphics: What is the quality and resolution of the graphical elements used for buttons, icons, menus, and content?	30	3	5	4.57	0.690
12	Visual Appeal: How visually appealing is the app overall?	30	3	5	4.20	0.664
	**Information Quality Domain**					
13	Information Quality: Is the app content correct, well written, and relevant to the app’s purpose/topic?	30	3	5	4.55	0.572
14	Quantity of Information: Is the information provided complete and objective?	20	1	5	4.10	1.012
15	Visual Information: Are visual explanations of concepts (tables, graphs, images, videos, etc.) clear, logical, and accurate?	30	3	5	4.54	0.637
16	Credibility: Does the information appear to come from a reliable and trustworthy source?	30	3	5	4.50	0.638
17	Recommendation: Would you recommend this app to people who could benefit from using it?	30	1	5	3.90	1.094
18	Intended Use: How often do you think you would use this app over the next 12 months if it were relevant to you?	30	1	5	3.70	1.179
	**Subjective Quality Domain**					
19	Would you pay for this app?	30	1	5	2.30	1.055
20	What is your overall star rating for the app?	30	2	5	3.80	0.847
	**Perceived Impact Domain**					
21	Awareness: This app increased my awareness of the importance of addressing my health-related habits and behaviors.	30	2	5	4.03	0.718
22	Knowledge: This app increased my knowledge or understanding regarding my health-related habits and behaviours.	30	1	5	3.73	0.944
23	Attitudes: This app changed my attitudes in a way that improved my health-related habits and behaviours.	30	1	5	3.67	0.922
24	Intention to Change: This app increased my intention or motivation to address my health-related habits and behaviours.	30	1	5	3.90	0.885
25	Help Seeking: This app would encourage me to seek additional help to deal with my health-related habits and behaviors (if needed).	30	1	5	3.93	0.868
26	Behaviour Change: Using this app will increase or decrease my health-related habits and behaviours.	30	1	5	3.83	0.950

**Table 3:** Summary of mean scores by domain and qualitative classification (uMARS-PT).

Domain	Items	Mean (Domain)	Qualitative Rating
Engagement	1–5	3.71	Acceptable/Moderate Quality
Functionality	6–9	4.51	Excellent Quality
Aesthetics	10–12	4.45	Excellent Quality
Information Quality	13–18	4.22	Excellent Quality
Subjective Quality	19–20	3.05	Acceptable/Moderate Quality
Perceived Impact	21–26	3.85	Acceptable/Moderate Quality
Overall MARS Score	1–18	4.22	Excellent Quality

## Data Availability

The original contributions presented in this study are included in the article. Further inquiries can be directed to the corresponding author.

## Abbreviations

The following abbreviations are used in this manuscript:

GDPT	General Data Protection Regulation
MARS	Mobile App Rating Scale
uMARS-PT	User Mobile App Rating Scale—Version PT
N/A	Non-applicable
WHO	World Health Organization
